# Each day is a new beginning

**DOI:** 10.1186/s40248-017-0085-4

**Published:** 2017-02-15

**Authors:** Francesco Blasi, Claudio M. Sanguinetti

**Affiliations:** 1 Department of Pathophysiology and Transplantation, Università degli Studi di Milano, Cardio-thoracic Unit and Cystic Fibrosis Adult Center Fondazione IRCCS Cà Granda Ospedale Maggiore Policlinico Milano, Milan, Italy; 2 Respiratory Medicine, Quisisana Clinical Center, Pneumology and Respiratory Intensive Care Unit, S.Filippo Neri General Hospital, Rome, Italy

*Multidisciplinary Respiratory Medicine* (*MRM*) is changing, as new Editors-in Chief we are pleased to introduce the new *MRM* to our readers and authors.

*MRM* history dates back to 2006, still a young Journal but with the aim to become a major player in the scientific field of respiratory diseases.

Since then, *MRM* has taken long strides towards even more prestigious goals and it holds now a distinguished position among the scientific respiratory journals.

In 2016 *MRM* has become the official journal of the Italian Respiratory Society (SIP/IRS) and according to the vision of SIP/IRS will address the different aspects of respiratory diseases with an interdisciplinary approach, aiming to fulfil the gap between specialists of different disciplines in the management and treatment of the respiratory patient.

The new beginning of our Journal starts from a new and dedicated Editorial Board that will help us to define a new profile of *MRM* with new sections and new editorial initiatives with a special attention to a further improvement of the selection of papers.

We plan to add a section dedicated to *Research Letters* that will cover papers with promising data, preliminary results and pivotal studies, with a special attention to young researchers works.

Furthermore, we are preparing a list of special issues of the Journal dedicated to specific hot topics with *ad hoc* Editors who will be responsible of the single issue that will include review articles and research papers.

Finally, *MRM* will publish thematic series, commissioning manuscripts from high level researchers in different specialties to underline the multidisciplinary vision of *MRM*.

We realise that an open access journal has some advantages, e.g. papers accessible online immediately upon publication without subscription charges or registration barriers, and some disavantages, e.g. article processing charges.

We are working together with the SIP/IRS to support the processing charges with the aim to promote the publication of high level papers from young researchers.

Any new beginning requires intensive efforts but we are confident that with the enthusiasm of our new Editorial Board, the great dedication of our publisher and the support of our readers and authors, *MRM* will reach its aims and a new important standing in the panorama of scientific publications.

